# TGF-β1 Induces Polypyrimidine Tract-Binding Protein to Alter Fibroblasts Proliferation and Fibronectin Deposition in Keloid

**DOI:** 10.1038/srep38033

**Published:** 2016-11-29

**Authors:** Hu Jiao, Ping Dong, Li Yan, Zhigang Yang, Xiaoyan Lv, Qiuchen Li, Xianlei Zong, Jincai Fan, Xin Fu, Xia Liu, Ran Xiao

**Affiliations:** 1 Research Center of Plastic Surgery Hospital, Chinese Academy of Medical Sciences & Peking Union Medical College, Beijing, P.R. China; 2 Scar Plastic Department of Plastic Surgery Hospital, Chinese Academy of Medical Sciences & Peking Union Medical College, Beijing, P.R. China

## Abstract

Human dermal fibrotic disease keloid has been a clinical challenge because of its tumour-like growth and the lack of effective therapy. Dysregulated alternative splicing events have been demonstrated in tumours and fibrosis. In the current study, for the first time, it was demonstrated that the splicing regulator polypyrimidine tract-binding protein (PTB), which plays a pivotal role in tumour proliferation, invasion and metastasis, is overexpressed in keloid tissues and fibroblasts. Additionally, TGF-β1 upregulated the expressions of PTB and its upstream regulator, C-MYC, in keloid fibroblasts. Furthermore, we suppressed PTB using siRNA in keloid fibroblasts and in a keloid xenograft nude mouse model. PTB knockdown significantly slowed the proliferation of keloid fibroblasts and accelerated the regression of transplanted keloid tissues, which was accompanied by a shift in the alternative splicing of USP5 and RTN4. Moreover, when PTB was suppressed, there was a reduction in excessive deposition of FN1 and COL3A1 in transplanted keloid tissues. However, only FN1 was downregulated in keloid fibroblasts that were cultured in media supplemented with TGF-β1. Our study provides evidence for the role of PTB in keloid pathophysiology and offers a novel therapeutic target for keloids. Most importantly, the role TGF-β1 regulation of PTB may provide new insights into the mechanisms underlying inflammatory cytokine-induced fibrosis.

Keloid is a fibrotic skin disease with the primary pathological feature of excessive extracellular matrix (ECM) deposition in lesions following increased proliferation of dermal fibroblasts^1^. Keloid is also regarded as a benign skin tumour because of its similarity to tumours in clinical features and pathological characteristics, such as invasion of normal tissues and recurrence despite treatments^2^,^3^, as well as increased cell proliferation and high growth factors status accompanied by uncontrolled growth. A meta-analysis showed that most treatments for keloids offer a minimal likelihood of improvement^4^, and its high recurrence rates make keloids one of the major unsolved clinical challenges in wound healing^5^. Gene silencing using siRNA is considered a potential promising therapeutic approach for human diseases with likely high specificity and potency^6^. Moreover, local administration of siRNA has become an attractive and effective route of application because of easy accessibility to the affected areas, reduced systemic effects, avoidance of first-pass metabolism, and ease of medication administration. All these factors make skin disorders a particularly suitable human disease model to benefit from siRNA therapy^7^. Therefore, the strategy of using siRNA to suppress the proliferation of keloid fibroblasts and subsequent ECM accumulation may be an alternative treatment for keloids, however, finding an effective target gene is the key to the success of this therapy. There are very limited *in vitro* studies on the use of siRNA transduction as a method to increase apoptosis of keloid fibroblasts or decrease ECM production by targeting phenotype-related genes^8^,^9^. However, keloid is a complex disease involving multiple events, and thus, siRNA treatment could be more effective if more upstream pathogenesis-related genes are targeted.

Alternative splicing allows the production of multiple mRNA variants and downstream proteins from one single gene via the inclusion or exclusion of specific exons^10^, and is generally regulated by *cis*-acting splicing sequences in primary transcripts and *trans*-acting splicing factors that bind to these RNA sequences. This process occurs in 95% of all multi-exonic genes^11^, and the dysregulation of alternative splicing plays diverse roles in numerous human diseases^12^,^13^. Aberrant alternative splicing contributes to many cancer phenotypes, therefore, the therapeutic intervention targeting either the cancer-specific alternative splicing events themselves or the splicing factors that dysregulate them is very promising. In our previous study, we reported that altered FGF-FGFR2 signalling pathways caused by abnormal alternative splicing of the *FGFR2* gene influenced the cellular phenotype in keloids^14^. As specific alterations in the expression of splicing factors in numerous diseases have been demonstrated, we also screened relevant splicing regulators for FGFR2 in keloid tissues and fibroblasts and found increased expression of polypyrimidine tract-binding protein (PTB), a negative splicing regulator of the FGFR2-IIIb isoform, that effects its function by binding to the silencing elements around exon IIIb^15^. PTB, also known as p57 and heterogeneous nuclear ribonucleoprotein I^16^,^17^, is a widely expressed RNA binding protein that is differentially expressed in different tissues and cells^18^. Several studies have reported the involvement of PTB in the regulation of tumour cell proliferation and decreased growth of tumor cells in PTB knockdown models^17^,^18^,^19^. PTB has been considered a potential therapeutic target for tumours, and a patent on PTB siRNA was submitted for the treatment of ovarian cancer and breast cancer^20^. We hypothesized that PTB may play a profound role in keloids development and could be a potential therapeutic target for keloids.

In the current study, PTB was found to be enriched in keloid tissues and fibroblasts as well as in keloid fibroblasts treated with transforming growth factor (TGF)-β1. To explore the function of PTB in keloid pathogenesis and evaluate its potential as a therapeutic target for the treatment of keloids, we suppressed the expression of PTB using siRNA in keloid fibroblasts and keloid xenografts grown in a nude mouse model, and the alternative splicing of multiple genes involved in cell proliferation and the expressions of ECM genes were investigated.

## Results

### The accumulation of ECM and proliferation of dermal cells in keloids

**Figure 1:**
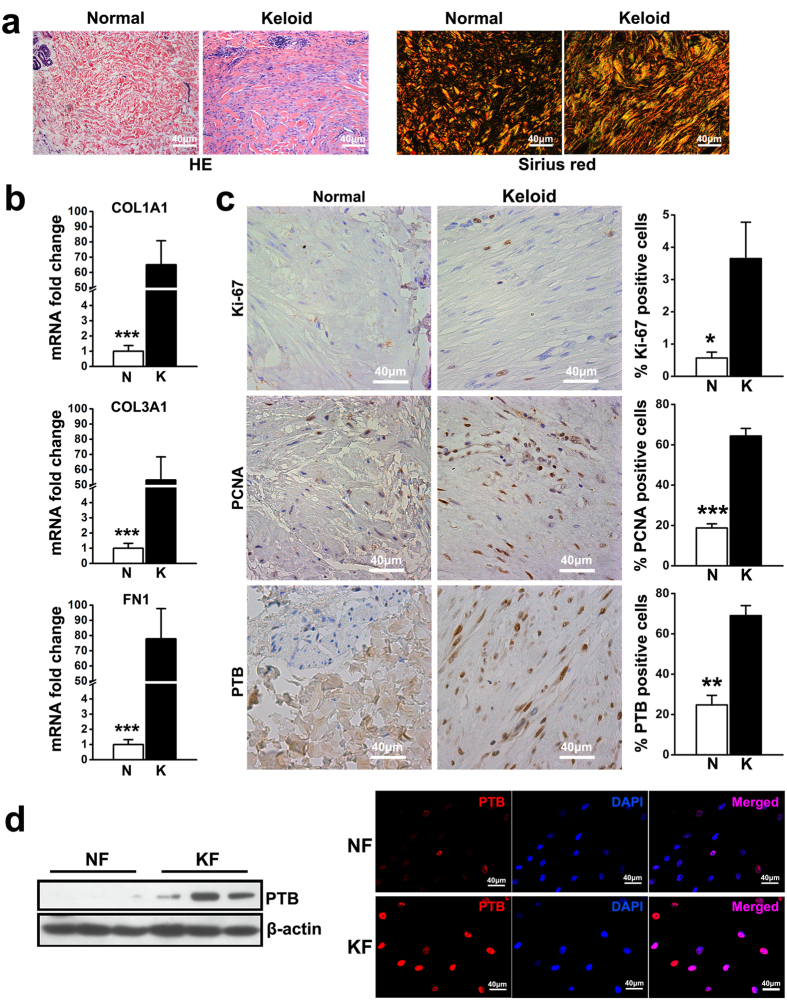
Accumulated ECM, increased cell proliferation and upregulated PTB expression were detected in keloid. (**a**) HE staining and sirius red staining were conducted for keloid tissues and normal skin. (**b**) The expression of *COL1A1*, *COL3A1* and *FN1* in dermal tissues was evaluated by real-time PCR. Data are shown as the mean ± SEM, n = 15. ***P < 0.001 by student’s t-test. (**c**) The expression of Ki-67, proliferating cell nuclear antigen (PCNA) and PTB was tested using immunohistochemistry analysis. Data are shown as the mean ± SD, n = 15. *P < 0.05, **P < 0.01, ***P < 0.001 by student’s t-test. (**d**) Passage three of fibroblasts were harvested from normal skin and keloid tissues to detect the expression of PTB by western blot and immunofluorescence, respectively. In western blot analysis the expression of β-actin was used as an internal control for protein loading normalization. N: normal skin; K: keloid tissue. NF: fibroblasts from normal skin; and KF: fibroblasts from keloid tissues.

HE staining in normal dermis showed thin collagen fibres and sparse spindle-shaped cells, whereas the keloid dermis was characterized by excessive accumulation of ECM with abnormally thick, hyalinised and compact collagen fibres as well as a large number of spindle-shaped cells. Sirius red staining showed that the levels of both collagens type I fibres (red and yellow) and type III fibres (green) increased in keloid dermis (Fig. 1a). Consistently, real-time PCR results showed that compared with normal dermis, the expression levels of collagen type I alpha1 (COL1A1), collagen type III alpha1 (COL3A1) and fibronectin 1 (FN1) were elevated by approximately 65-fold, 53-fold and 78-fold in keloid dermis, respectively (Fig. 1b).

Immunohistochemistry staining of the cell proliferation markers proliferating cell nuclear antigen and Ki-67 showed significantly higher expression levels in keloid dermis than those in normal dermis, suggesting increased proliferation of dermal cells in keloid tissues. The expression of PTB in dermal tissue was also observed and indicated that approximately 70% of cells were positive for PTB in keloid dermis, while only a few cells in normal dermis were PTB positive (Fig. 1c). Additionally, the results of western blot analysis and immunofluorescence staining showed an elevated expression of PTB in keloid fibroblasts from different specimens compared to dermal fibroblasts derived from normal skin (Fig. 1d).

### TGF-β1 treatment on ECM secretion and PTB expression in keloid fibroblasts

**Figure 2:**
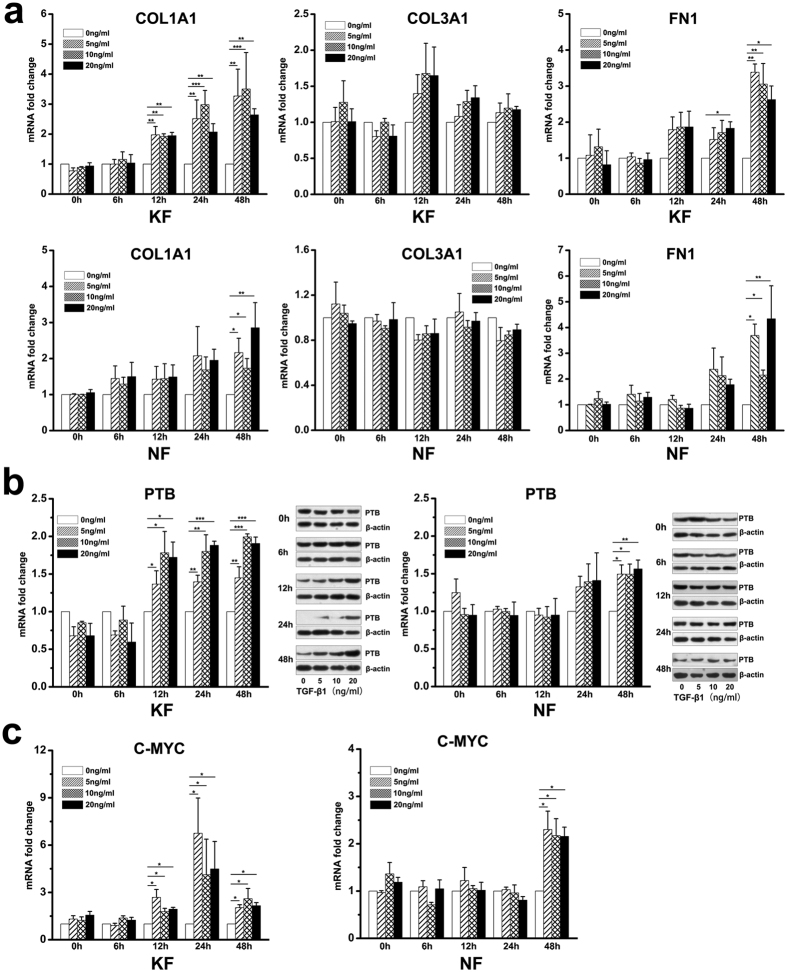
TGF-β1 treatment increased ECM secretion and PTB expression in fibroblasts from keloid and normal skin. Fibroblasts were cultured in medium containing different concentrations of TGF-β1 (0 ng/ml, 5 ng/ml, 10 ng/ml, 20 ng/ml) for assays at different time intervals (0 h, 6 h, 12 h, 24 h, 48 h). (**a**) The expression levels of ECM genes *COL1A1*, *COL3A1* and *FN1* were analysed by real-time PCR after TGF-β1 treatment. (**b**) PTB expression in fibroblasts after TGF-β1 treatment was tested at mRNA and protein levels. In western blot analysis, the expression of β-actin was used as an internal control for protein loading normalization, and the gels loaded with samples at different time points have been run under the same conditions. (**c**) The expression of *C-MYC* was evaluated by real-time PCR. Data are shown as the mean ± SEM, n = 4 for each time point. *P < 0.05, **P < 0.01, ***P < 0.001 by Least Significant Difference test. NF: fibroblasts from normal skin; KF: fibroblasts from keloid tissue.

We cultured both keloid and normal fibroblasts in medium supplemented with different concentrations of TGF-β1. After 12 hours of TGF-β1 treatment, the expression of COL1A1 was increase in keloid fibroblasts, while in normal fibroblasts the increase was observed after 48 hours. The expression of FN1 increased significantly after 24 hours of TGF-β1 treatment in keloid fibroblasts, which was earlier than in normal fibroblasts. However, the expression of COL3A1 was not altered after TGF-β1 treatment in both normal and keloid fibroblasts (Fig. 2a).

The expression of PTB in keloid fibroblasts was observed to increase significantly after 12 hours of TGF-β1 stimulation at all the three concentrations tested. However, in normal fibroblasts TGF-β1 caused a weaker effect on the expression of PTB that remained unchanged after 48 hours of treatment. Changes in the expression of PTB were confirmed by western blot analysis, which showed that the expression began to increase after 12 hours of TGF-β1 treatment in keloid fibroblasts, while in normal fibroblasts, increases was observed after 48 hours (Fig. 2b). The regulation of PTB expression by TGF-β1 has only been described in a few reports in the literature^21^. To give a hint for the mechanism by which TGF-β1 regulates PTB, the expression of C-MYC, which binds to the PTB promoter and then upregulates the expression of PTB^22^, was analysed in dermal fibroblasts that were treated with TGF-β1. In both keloid and normal fibroblasts, the expression of C-MYC showed a similar increasing trend as PTB (Fig. 2c). Our findings imply that in keloids, PTB may be upregulated by TGF-β1 through C-MYC.

### Effect of knockdown of PTB on the proliferation of keloid fibroblasts

**Figure 3:**
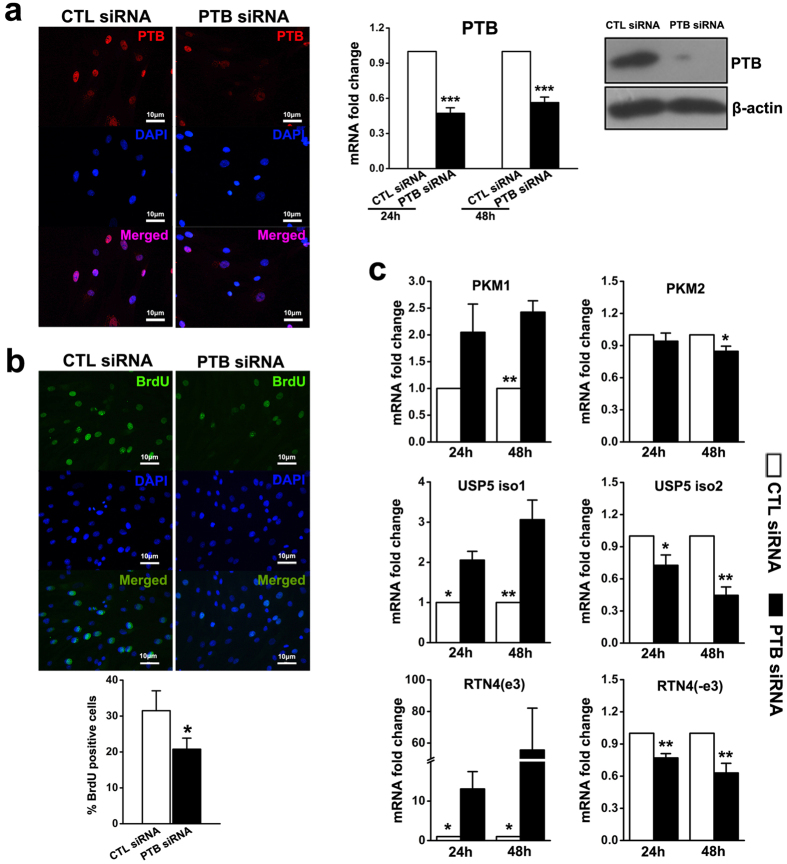
*In vitro* PTB siRNA decreased the proliferation of keloid fibroblasts with alternative splicing switching of genes involved in the cell proliferation. (**a**) Keloid fibroblasts were transfected with PTB siRNA or control siRNA, and the expression of PTB was analysed by immunofluorescence, real-time PCR and western blot post transfection. Data are shown as the mean ± SEM, n = 4. ***P < 0.001 by paired student’s t-test. (**b**) Proliferation of keloid fibroblasts after PTB knockdown was detected by BrdU analysis. Data are shown as the mean ± SD, n = 4. *P < 0.05 by paired student’s t-test. (**c**) The expression levels of genes involved in cell proliferation were analysed by real-time PCR. Data are shown as the mean ± SEM, n = 4. **P < 0.01, ***P < 0.001 by paired student’s t-test. CTL siRNA: Control siRNA group.

SiRNA against PTB was used to suppress PTB expression in keloid fibroblasts. At both 24 and 48 hours post transfection, keloid fibroblasts showed over 50% less PTB mRNA compared to the control siRNA transfected group. Immunofluorescence staining and western blot analysis confirmed the significant inhibition of PTB expression by siRNA (Fig. 3a). BrdU cell proliferation assay showed a decrease in BrdU positive keloid fibroblasts in the PTB siRNA group compared to the control siRNA group (Fig. 3b). Because PTB plays a pivotal role in the alternative splicing of multiple genes, including some genes involved in the proliferation of tumour cells, such as pyruvate kinase, ubiquitin-specific protease 5 (USP5) and reticulon-4 (RTN4), we analysed changes in the expression of different alternative splicing isoforms of these genes after PTB knockdown. As expected, mRNA expression of pyruvate kinase M1 isoform (PKM1) increased by more than 2-fold 48 hours after transfection with PTB siRNA. Conversely, pyruvate kinase M2 isoform (PKM2) mRNA decreased significantly 48 hours after transfection. Moreover, PTB knockdown shifted the splicing of USP5 from isoform 2 to isoform 1, and the splicing of RTN4 from the isoform excluding exon 3 to the isoform including exon 3 (Fig. 3c). To rule out off-target effects, we used another siRNA sequence of PTB and observed similar results (data not shown).

### Knockdown of PTB on ECM secretion of keloid fibroblasts

**Figure 4:**
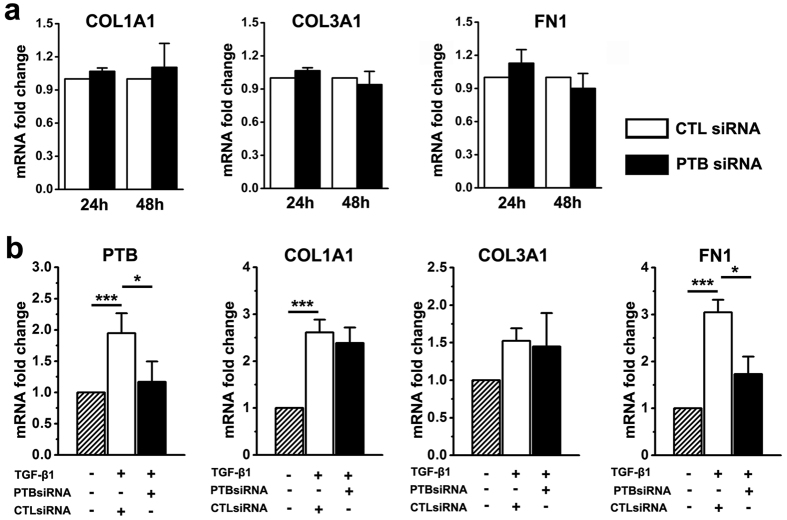
PTB knockdown reduced the expression of FN1 in TGF-β1-treated keloid fibroblasts. (**a**) The expression levels of genes encoding ECM of keloid fibroblasts cultured in normal medium were examined using real-time PCR post PTB knockdown. (**b**) Keloid fibroblasts were also transfected with PTB siRNA in medium containing TGF-β1, and the expression levels of *PTB* and the genes encoding ECM were analysed by real-time PCR at 48 hours post transfection. Data are shown as the mean ± SEM, n = 4. *P < 0.05, ***P < 0.001 by Least Significant Difference test. CTL siRNA: Control siRNA group.

At both 24 and 48 hours post transfection with PTB siRNA, keloid fibroblasts did not show any significant change on the mRNA expression levels for ECM genes *COL1A1*, *COL3A1* and *FN1*, indicating that PTB may not regulate the expressions of ECM genes in keloid fibroblasts under regular culture conditions (Fig. 4a). However, when keloid fibroblasts were treated by TGF-β1 for 48 hours after PTB siRNA and control siRNA transfection, TGF-β1 increased the expressions of COL1A1 and FN1 without changing COL3A1 in the control siRNA group, whereas the expression of FN1 was significantly lower in the PTB siRNA group than in the control siRNA group (Fig. 4b). Similar results were found when PTB knockdown was created using a different siRNA (data not shown). These results imply that PTB is involved in TGF-β1 induction of fibronectin secretion in keloid fibroblasts.

### PTB siRNA injection in a keloid xenograft nude mouse model

**Figure 5:**
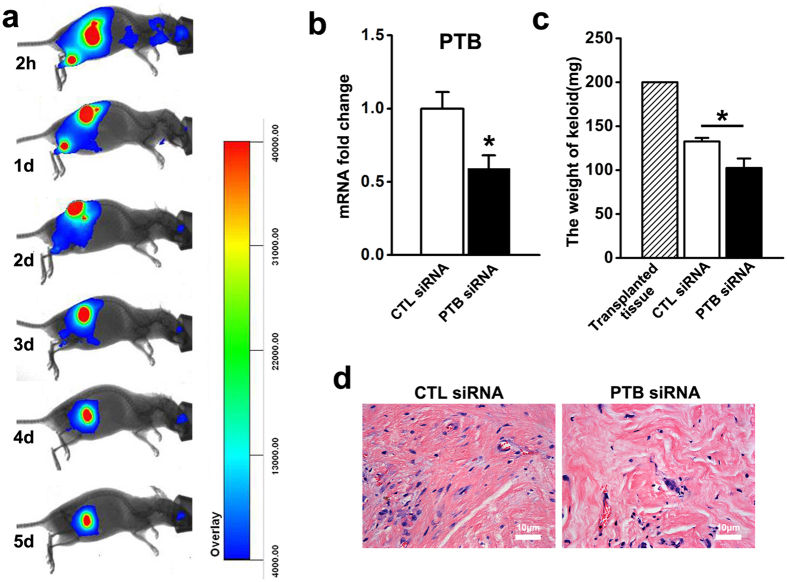
*In vivo*, PTB siRNA reduced the expression of PTB and accelerated the regression of xenografted keloid tissues. (**a**) Keloid xenograft mouse model was established, Cy5-siRNA was injected into xenografted keloid tissue and the fluorescence signal was detected by whole-body fluorescence imaging at 2 hours, 1 day, 2 days, 3 days, 4 days, 5 days after injection. (**b**) After siRNA injection for four weeks, transplanted keloid tissues were harvested, and the expression of PTB was detected by real-time PCR. Data are shown as the mean ± SEM, n = 16. *P < 0.05 by student’s t-test. (**c**) The weights of transplanted keloid tissues was measured. Data are shown as the mean ± SD, n = 16. *P < 0.05 by student’s t-test. (**d**) HE staining was conducted for the xenografted keloid tissues. CTL siRNA: Control siRNA group.

We established a keloid xenograft nude mouse model and examined the effect of PTB siRNA injection on the growth of tumours. The pharmacokinetics of siRNA in xenografted keloid tissues revealed that the siRNA fluorescence signals decreased by 50% after three to four days (Fig. 5a). We injected PTB siRNA or control siRNA into xenograft keloid tissues twice a week. After eight injections over four weeks, the transplanted keloid tissues were excised for analysis. The expression of PTB was evaluated and showed that PTB siRNA reduced the expression of PTB *in vivo* (Fig. 5b). The weights of transplanted tissues in both PTB and control siRNA groups were lighter than those prior to the transplantation; however, the weights decreased more in PTB siRNA group (Fig. 5c). HE staining showed less cells density and collagen fibre content in the PTB siRNA group (Fig. 5d).

**Figure 6:**
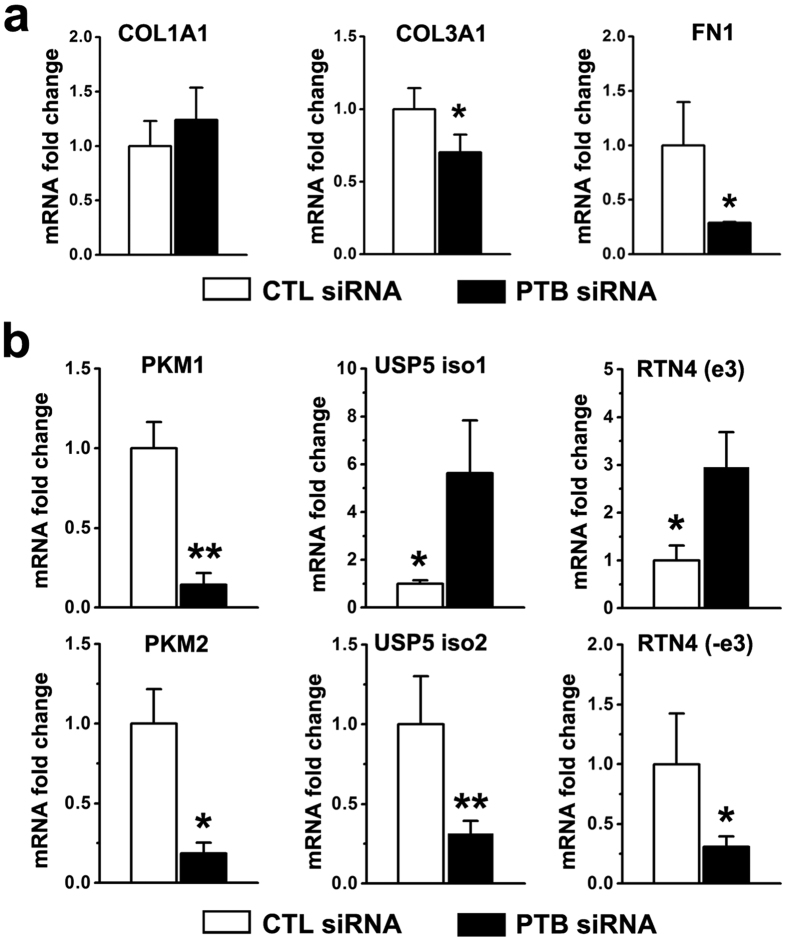
*In vivo*, PTB siRNA reduced the expressions of ECM genes and influenced the alternative splicing of genes involved in cell proliferation. (**a**) The expression levels of *COL1A1*, *COL3A1* and *FN1* were evaluated by real-time PCR. (**b**) Alternative splicing isoforms of genes involved in cell proliferation were evaluated by real-time PCR. Data are shown as the mean ± SEM, n = 16. *P < 0.05, *P < 0.05, **P < 0.01 by student’s t-test. CTL siRNA: Control siRNA group.

The expressions of genes involved in cell proliferation and ECM accumulation were evaluated in keloid xenograft tissues. In the PTB siRNA group, the expressions of *COL3A1* and *FN1* but not *COL1A1* significantly decreased in keloid xenograft tissues (Fig. 6a). Meanwhile, in the PTB siRNA group, the expression of both *PKM1* and *PKM2* decreased, USP5 isoform 1 increased, and exon 3 inclusion of RTN4 was enhanced with a concomitant decrease of USP5 isoform 2 and RTN4 without exon 3 (Fig. 6b). The results may explain the lower cell density and ECM accumulation in the PTB siRNA group, and suggest that PTB regulates cell proliferation and transcription of *COL3A1* and *FN1 in vivo* in keloid tissues.

## Discussion

Treatment of keloid has been a significant challenge to surgeons, as its precise pathogenesis still remains unclear. Splicing regulators are the major players in proteome diversity in humans and thus are very relevant to diseases and therapies. Our previous study showed that the epithelial-mesenchymal transition was concomitant with the alternative splicing of FGFR2 IIIb/IIIc in keloid tissues^14^. In the current study, the FGFR2 splicing regulator PTB, whose overexpression plays a pivotal role in tumour proliferation, invasion and metastasis, is demonstrated for the first time to be overexpressed in keloid tissues and fibroblasts. Moreover, fibrosis cytokine TGF-β1 was found to promote ECM secretion and the expression of PTB and C-MYC in keloid fibroblasts. PTB knockdown significantly slowed the proliferation of keloid fibroblasts by altering the alternative splicing of genes involved in cell proliferation, and decreased the expression of TGF-β1-induced FN1. Furthermore, we established a keloid xenograft nude mouse model and knocked down PTB using an siRNA injection approach. The *in vivo* results showed that PTB siRNA accelerated keloid tissue regression through regulating both dermal cell proliferation and ECM accumulation. Our study provides evidence of the role of PTB in keloid pathophysiology and offers a novel therapeutic target for keloids.

TGF-β1 is the most well-known and important cytokine for keloids initiation and contributes to fibrosis formation in response to tissue injury in a number of organs, including lung, liver, pancreas, and kidney^23^. Our data demonstrated that the expression levels of COL1A1 and FN1 in keloid fibroblasts were significantly elevated after TGF-β1 treatment, which is consistent with other reports in the literature^24^,^25^,^26^. Moreover, we first reported the expression of PTB and its upstream regulator C-MYC showed the similar increasing trend in keloid fibroblasts upon TGF-β1 induction. Indeed, Hallgren^21^ reported changes in the expression of a total of 1733 proteins in human lung fibroblasts following treatment with TGF-β1. One of these proteins was PTB, but this result has not been further explored. The oncogenic transcription factor C-MYC is overexpressed in a broad range of tumours and has been shown to bind to the PTB promoter^27^,^28^ and upregulate the expression of PTB^22^,^29^,^30^. Our findings indicate that PTB may be upregulated by TGF-β1 through C-MYC in keloids, a link which could be a potential pathogenic mechanism for fibrotic disease.

PTB plays multiple roles in the synthesis and maturation of mRNA, including the alternative splicing of pre-mRNA, 3′-end polyadenylation of pre-mRNA and internal ribosome entry site-dependent translation, and it also functions in the transportation and stabilization of mRNA in the cytoplasm^31^,^32^,^33^,^34^. It was reported that the expression of PTB is often increased in cancer cells, such as in human epithelial ovarian tumour, glioblastoma, prostate cancer, cervical cancer, neuroblastoma, multiple myeloma and osteosarcoma^17^,^19^,^35^,^36^. The alternative splicing regulated by PTB has been demonstrated to be involved in tumour cell proliferation in addition to the two mutually exclusive alternative splicing isoforms of pyruvate kinase^22^,^37^,^38^. PKM2 promotes aerobic glycolysis to support cell growth and is predominantly expressed in tumor cells, while PKM1 is the dominant isoform in normal cells. Modulation of USP5 splicing generates a shorter isoform 2 without exon 15 that is strongly correlated with cell proliferation and overexpressed in glioblastoma^39^, whereas USP5 isoform 1 with exon 15 is associated with reduced cell growth and motility in glioblastoma. In human glioma cells, depletion of PTB slowed cell proliferation with enhanced inclusion of exon 3 of RTN4, and the overexpression of RTN4 splicing isoform with exon 3 resulted in a similar degree of decreased cell proliferation as the removal of PTB^19^. Although a number of studies have mentioned increased proliferation in keloids^40^, the precise underlying mechanism is still unknown. Our data have demonstrated that inhibited proliferation of keloid fibroblasts due to the suppression of PTB is accompanied with the alternate splicing of USP5 and RTN4, which may be correlated to over proliferation of fibroblasts in keloid.

In the current study, PTB suppression also caused the reduction of FN1 expression in transplanted keloid tissues and TGF-β1-treated keloid fibroblasts. FN1 plays a major role in cell adhesion, migration and differentiation^41^, and its altered expression has been associated with cancer and fibrosis^42^,^43^,^44^. Fibronectin was found to be strongly expressed in both hypertrophic and keloid tissues compared with normal dermis, and localized to the fibrotic lesions concerned with abnormal scarring mechanisms^45^. Moreover, it was shown that fibronectin expression was more responsive to the upregulation stimulated by TGF-β1 in keloid fibroblasts than in normal fibroblasts^46^. Knockdown of PTB decreased FN1 expression only in cells that were cultured in medium containing TGF-β1, implying that PTB may play a special role induced by TGF-β1 during fibrosis formation, an observation which has not been described previously. Moreover, COL3A1 was reduced in transplanted keloid tissues with PTB suppression, although its expression had not been changed after TGF-β1 treatment and PTB suppression *in vitro*. Our finding suggests that other signaling mechanisms may be activated to decrease COL3A1 through PTB *in vivo*, which needs further investigation.

## Methods

### Human tissue specimens

A total of 65 skin specimens including normal skin (NS, n = 30), and keloid (K, n = 35) were selected for the study. Keloid specimens were harvested from typical keloid patients at the time of surgical excision, while normal skin specimens were obtained from patients who underwent surgical procedures for cosmetic reasons and displayed no keloid, hypertrophic scars or current infections. Additionally, keloid had not been subjected to any type of therapy before surgical excision with an age ranged from 6 months to 5 years. All procedures in this study were approved by the Ethics Committee of the Plastic Surgery Hospital and all patients provided written informed consent. All experiments were performed in accordance with relevant guidelines and regulations.

### Histochemistry and immunohistochemistry

Biopsy specimens were placed in 10% formalin saline for 24 hours and dehydrated by standard histological procedures. The biopsies were embedded in paraffin, cut using a microtome (5 μm sections), and mounted on glass slides. The slides were stained with haematoxylin and eosin, and sirius red staining were performed according to the manufacturer’s instructions (MUTO, Tokyo, Japan). For immunohistochemistry staining, mouse monoclonal antibodies directed against PCNA (Zhongshan, Beijing, China), Ki-67 (Zhongshan, Beijing, China), and PTB (Abcam, HK, China) were used and horseradish peroxidase-conjugated rabbit anti-mouse IgG (Abcam, HK, China) acted as the secondary antibody. Staining was achieved using a DAB Stain kit (Zhongshan, Beijing, China). The number of positive cells was counted in five random high power fields (400× total magnification).

### Cell culture

Keloid and normal skin specimens were cut into 3 × 15 mm pieces and incubated in dispase (5 mg/ml) overnight at 4 °C. The epidermis was then peeled off with forceps the following day. The remnant dermis was minced and incubated in a solution of collagenase type I (0.5 mg/ml) at 37 °C for 5 hours. Cells were pelleted and grown in regular medium (Dulbecco’s modified Eagles’s medium (DMEM), supplemented with 10% fetal bovine serum (FBS), 100 U/ml penicillin and 100 ng/ml streptomycin). Fibroblasts from the third passage were used for the experiments.

### BrdU assay and PTB immunofluorescence staining

Cell proliferation was determined by measuring the incorporation of 5-bromo-2-deoxyuridine (BrdU) into DNA using BrdU Cell Proliferation Assay kit (Roche, Basel, Switzerland). The fibroblasts were grown on coverslips for 48 h and BrdU (10 mM) was added two hours before the cells were fixed in methanol-acetone (70%/30% v/v) for immunostaining according to the manufacturer’s instruction. For PTB immunofluorescence staining, the fibroblasts were fixed in 4% paraformaldehyde for 20 minutes at room temperature, then permeabilised in 0.1% Triton X-100 for 5 minutes, and blocked for 1 hour with 5% goat serum. The cells were then incubated overnight at 4 °C with primary antibodies targeting PTB (Abcam, HK, China) and developed with Alexa Fluor^®^ 488 donkey anti-mouse IgG (Thermo, Rockford, IL, USA). The slides were mounted with a solution containing DAPI (Zhongshan, Beijing, China) and imaged with a Leica DM3000 microscope (Leica, Solms, Germany).

### SiRNA transfection

Cells were transient transfected using siRNA transfection reagent (Santa Cruz, Dallas, TX, USA) with PTB-specific siRNA (siRNA targeted to *PTB* gene) or control siRNA (siRNA which is not directed against any target gene) synthesized by the RIBOBIO company (Guangzhou, China). The sequences of PTB-specific siRNA are as following: forward, 5′-GCAAGAAGUUCAAAGGUGAdTdT-3′; reverse, 3′-dTdT CGUUCUUCAAGUUUCCACU-5′. Briefly, the day before the transfection, 1 × 10^5^ fibroblasts per well were plated onto 12-well plates, and the siRNA was transfected with siRNA transfection reagent to each well when the cells had reached 30% to 50% confluence. After 8 hours of transfection, the medium was replaced by the regular medium which was defined as 0 hours post-transfection. Fibroblasts were incubated in the regular medium for 24 h or 48 h before experiments. The efficiency of siRNAs was evaluated by real-time PCR and western blot. We also tried another siRNA sequence of PTB (Santa Cruz, Dallas, TX, USA) for *in vitro* knockdown experiment.

### TGF-β1 treatment

The fibroblasts were plated in 12-well plates and incubated in regular medium. When cells reached 80% confluence, the regular medium was replaced by DMEM to starve cells. After 24 hours of starving, the medium was replaced by DMEM containing different concentrations of recombinant human TGF-β1 (0 ng/ml, 5 ng/ml, 10 ng/ml, 20 ng/ml, PeproTech, Rocky Hill, NJ, USA). At different time intervals (0 hour, 6 hours, 12 hours, 24 hours, 48 hours) with TGF-β1 treatment, the fibroblasts were harvested for further assays. For those cells transfected with PTB siRNA or control siRNA, after 48 hours of TGF-β1 (20 ng/ml) treatment, the fibroblasts were harvested for further experiments.

### Keloid xenograft mouse model

We developed a keloid xenograft mouse model to test the efficacy of PTB siRNA *in vivo*. Animal experiments were approved by Institutional Animal Care and Use Committee of Plastic Surgery Hospital (Institute), and all methods were performed in accordance with the relevant guidelines and regulations. The dermal tissues of keloid were cut into approximately 0.4 × 0.5 × 1.0 cm^3^ sections, and the weight was 200 mg per tissue block. Then, the nude mouse was anesthetized, a cut approximately 0.5 cm was made into the dorsal skin, a subcutaneous dissection was made to form a pocket, and a tissue block was implanted into the subcutaneous pocket. After 14 days of implantation, the mice were ready for siRNA knockdown. For each transplant, siRNA was dissolved into 50 μl PBS at a concentration of 0.8 μg/ml and injected slowly with a 23 G syringe needle. To determine the half-life and the proper injection intervals, Cy5-siRNA was injected into the transplants, and the fluorescence signal was detected by whole-body fluorescence imaging technique at 2 hours, 1 day, 2 days, 3 days, 4 days, 5 days after injection. According to the whole-body imaging findings, the mice were injected PTB siRNA or control siRNA, twice a week for four weeks. The weight of the transplants was determined at the time of excision after 4 weeks of siRNA treatment, and further analysis of histology and gene expressions were performed.

### RNA extraction and real-time PCR

Total RNA was isolated from tissue or cultured cells using TRI Reagent (Sigma, St. Louis, MO, USA) according to the manufacturer’s instructions. Reverse transcription was conducted with 500 ng RNA using a reverse transcript cDNA synthesis kit (Promega, Madison, WI, USA). To quantify the transcripts of genes of interest, real-time PCR was performed using a SYBR Green Premix Ex Taq (KAPA, Woburn, MA, USA) on LightCycler 480II (Roche, Basel, Switzerland). Specifically, 1 μl of reverse transcription reaction mixture was utilized for a quantitative PCR reaction in a total volume of 20 μl. Relative expression levels of target genes= 2-rrCt (rrCt=rCt (experimental group) - rCt (control group)). B2M (beta 2 microglobin) was used as an internal reference control. Primers used for real-time PCR amplification are listed in Table 1. For gene expression analyses, the values were normalized to control group, indicated as 1 and the mRNA fold changes were calculated.

### Western blot analysis

Total protein extracted from normal skin and keloid lesions were prepared using the Total Protein Extraction Kit (Applygen, Beijing, China). Protein concentrations were determined using a BCA Protein Assay kit (Solarbio, Beijing, China). Western blotting was carried out using the antibodies for anti-PTB (1:1500 dilution; Abcam, HK, China) and anti-β-actin (1:5000 dilution; Abcam, HK, China), which was used as an internal control for protein loading normalization.

### Statistical analysis

Statistical analyses were performed using the SPSS version 16.0 (SPSS, Chicago, USA). Data were shown as the mean ± SEM (standard errors of the mean) or mean ± SD (standard deviation). Data involving only two groups was analysed using a two-tailed student’s t-test. When more than two experimental groups were compared, the data were analysed using the Least Significant Difference test to compare data between individual experimental groups. The level of statistical significance was set at P < 0.05.

## Additional Information

**How to cite this article**: Jiao, H. *et al*. TGF-β1 Induces Polypyrimidine Tract-Binding Protein to Alter Fibroblasts Proliferation and Fibronectin Deposition in Keloid. *Sci. Rep.* **6**, 38033; doi: 10.1038/srep38033 (2016).

**Publisher's note:** Springer Nature remains neutral with regard to jurisdictional claims in published maps and institutional affiliations.

## Figures and Tables

**Table 1:** Primers used for real-time PCR.

Genes	Primers	
B2M	forward	ACTGAATTCACCCCCACTGA
	reverse	CCTCCATGATGCTGCTTACA
PTB	forward	ACGCACATTCCGTTGCCTTAC
	reverse	AACCTGCCTCTACAGCGTCCA
COL1A1	forward	CCCGGGTTTCAGAGACAACTTC
	reverse	TCCACATGCTTTATTCCAGCAATC
COL3A1	forward	ATGAAGGTGAATTCAAGGCTGAAG
	reverse	CCACCAATGTCATAGGGTGCAATA
FN1	forward	CTAGGCAATGCGTTGGTTTGTA
	reverse	TGTCACCCACTCGGTAAGTGTTC
USP5(isoform1)	forward	CTATGGCAACGAAGACGAAGA
	reverse	CTGTTGCCCGTGTAGTAGAC
USP5(isoform2)	forward	TGAGCCCAAAGCGCCCATG
	reverse	GCTGTTGCCCGTGTAGTAGAC
PKM1	forward	CTTTGATAGTTCTGACGGAG
	reverse	CAGGGAAGATGCCACGGTA
PKM2	forward	TGCCGTGGAGGCCTCCTTCAAGT
	reverse	GGGGCACGTGGGCGGTATCTG
RTN4(e3)	forward	AAGATACCCTGTTACCTGATG
	reverse	CTCTTATCTGTGCTTCCTTAG
RTN4(-e3)	forward	AACAGCCTACATTGCCTTGG
	reverse	GCCTGAGTTCCTTTATCGTG
